# Characterization of synthetic wheat line Largo for resistance to stem rust

**DOI:** 10.1093/g3journal/jkab193

**Published:** 2021-06-04

**Authors:** Jyoti Saini Sharma, Megan Overlander, Justin D Faris, Daryl L Klindworth, Matthew N Rouse, Houyang Kang, Yunming Long, Yue Jin, Evans S Lagudah, Steven S Xu

**Affiliations:** 1 Department of Plant Sciences, North Dakota State University, Fargo, ND 58108, USA; 2 Cereal Crops Research Unit, Edward T. Schafer Agricultural Research Center, United States Department of Agriculture-Agricultural Research Service, Fargo, ND 58102, USA; 3 Cereal Disease Laboratory, United States Department of Agriculture-Agricultural Research Service and Department of Plant Pathology, University of Minnesota, Saint Paul, MN 55108, USA; 4 Triticeae Research Institute, Sichuan Agricultural University, Sichuan 611130, China; 5 Agriculture Flagship, Commonwealth Scientific and Industrial Research Organization, Canberra, ACT 2601, Australia

**Keywords:** synthetic hexaploid wheat, durum, *Aegilops tauschii*, Largo, stem rust, *Sr13*, *Sr46*

## Abstract

Resistance breeding is an effective approach against wheat stem rust caused by *Puccinia graminis* f. sp*. tritici* (*Pgt*). The synthetic hexaploid wheat line Largo (pedigree: durum wheat “Langdon” × *Aegilops tauschii* PI 268210) was found to have resistance to a broad spectrum of *Pgt* races including the Ug99 race group. To identify the stem rust resistance (*Sr*) genes, we genotyped a population of 188 recombinant inbred lines developed from a cross between the susceptible wheat line ND495 and Largo using the wheat Infinium 90 K SNP iSelect array and evaluated the population for seedling resistance to the *Pgt* races TTKSK, TRTTF, and TTTTF in the greenhouse conditions. Based on genetic linkage analysis using the marker and rust data, we identified six quantitative trait loci (QTL) with effectiveness against different races. Three QTL on chromosome arms 6AL, 2BL, and 2BS corresponded to *Sr* genes *Sr13c*, *Sr9e*, and a likely new gene from Langdon, respectively. Two other QTL from PI 268210 on 2DS and 1DS were associated with a potentially new allele of *Sr46* and a likely new *Sr* gene, respectively. In addition, *Sr7a* was identified as the underlying gene for the 4AL QTL from ND495. Knowledge of the *Sr* genes in Largo will help to design breeding experiments aimed to develop new stem rust-resistant wheat varieties. Largo and its derived lines are particularly useful for introducing two Ug99-effective genes *Sr13c* and *Sr46* into modern bread wheat varieties. The 90 K SNP-based high-density map will be useful for identifying the other important genes in Largo.

## Introduction

Wheat (*Triticum aestivum* L., 2*n* = 6*x* = 42, AABBDD) stem rust, caused by *Puccinia graminis* Pers.:Pers. f. sp*. tritici* Eriks & E. Henn (*Pgt*), is a major threat to wheat production worldwide. Since the *Pgt* race TTKSK (also known as Ug99) was first identified in Uganda in 1999, a total of 13 variants within the Ug99 lineage, commonly known as to the Ug99 race group, have been detected across 13 countries in East Africa and the Middle East over the last two decades (Pretorius *et al.* 2000; Singh *et al.* 2015; Patpour *et al.* 2016; Terefe *et al.* 2019). Li *et al.* (2019) recently proposed that high genetic diversity of Ug99 races largely resulted from somatic hybridization and nuclear exchange between dikaryons, which likely is a driving force for the emergence of new pathotypes in asexual fungal populations. In addition, there are a few non-Ug99 lineage *Pgt* races such as TRTTF, TTTTF, JRCQC, and TKTTF, known to carry virulence against frequently deployed stem rust resistance (*Sr*) genes such as *Sr9e*, *Sr25*, *Sr36*, *SrTmp*, and *Sr1RS^Amigo^* (Jin 2005; Jin and Singh 2006; Olivera *et al.* 2012, 2015; Olivera Firpo *et al.* 2017; Patpour *et al.* 2017). Olivera *et al.* (2019) also found different virulent combinations among the *Pgt* races collected from Georgia. Together these diverse *Pgt* races pose a serious threat to global food security. Development of resistant wheat varieties is an effective approach to counter these threats. To achieve this goal, the wheat research community continuously searches for new resistance genes.

The wheat primary gene pool has been considered the best resource of resistance (R) genes due to minimal deleterious effects caused by linkage drag. The hexaploid wheat D-genome progenitor *Aegilops tauschii* Coss. (2*n* = 2*x* = 14, DD) is known to be a great resource of R genes for various diseases and insect pests (Ogbonnaya *et al.* 2013; Arora *et al.* 2019). To utilize *Ae. tauschii* accessions for the development of the resistant wheat lines/cultivars against biotic stresses, Dr. Leonard R. Joppa (USDA-ARS, retired) developed over 40 synthetic hexaploid wheat (SHW) lines by crossing durum wheat [*T. turgidium* L. subsp. *durum* (Desf.) Husn., 2*n* = 4*x* = 28, AABB)] “Langdon” with different *Ae. tauschii* accessions (Xu *et al.* 2010). Among these Langdon-derived SHW germplasm, one line was released and named Largo (CI 17895), which carries the *Gb3* gene for greenbug (*Schizaphis graminum*, Rondani) resistance derived from *Ae. tauschii* accession PI 268210 (Joppa and Williams, 1982). Since its release, Largo and its derivatives have been the primary source of greenbug resistance in the winter wheat germplasm and varieties in Texas (Lazar *et al.* 1996, 1997; Rudd *et al.* 2014). Largo was also identified to carry resistance to wheat curl mite (*Aceria tosichella* Keifer) (Dhakal *et al.* 2018) and several fungal diseases, including Septoria tritici blotch (Adhikari *et al.* 2015), Fusarium head blight (Szabo-Hever *et al.* 2018), and stem rust (Friesen *et al.* 2008).

The durum wheat variety Langdon was developed using a modified backcross procedure to transfer stem rust resistance from Khapli emmer (*T. turgidum* subsp. *dicoccum* Schrank) during the stem rust outbreak of the 1950s in the Northern Great Plains (Heyne 1959). Previous studies indicated that Langdon carries at least four *Sr* genes (Salazar and Joppa 1981). However, besides *Sr13c* (Zhang *et al.* 2017; Gill *et al.* 2021), other *Sr* genes in Langdon have not been unambiguously identified and confirmed. Because Langdon is one of the founders of modern durum germplasm and varieties in the US, identification of the *Sr* genes it harbors will enhance our understanding of the *Sr* genes present in modern durum wheat germplasm. Similarly, *Ae*. *tauschii* accession PI 268210 was previously identified to be resistant to all *Pgt* races tested, including TTKSK (Friesen *et al.* 2008; Zhang 2013). However, the *Sr* gene(s) in PI 268210 has also not been identified.

In addition to its high value in bread wheat breeding, Largo should be a useful parental line of a permanent mapping population that can be used for identification, mapping, and marker development for the agronomically important genes derived from durum Langdon and *Ae. tauschii* PI 268210. We conducted this study intending to identify the genes controlling stem rust resistance by developing, genotyping, and phenotyping a recombinant inbred line (RIL) population from a cross between Largo and the bread wheat line ND495.

## Materials and methods

### Plant material and stem rust screening

A population of 226 RILs developed from a cross between a hard spring wheat line ND495 and SHW line Largo was used for genotypic and phenotypic analysis. Largo (CI 17895) was developed from a cross between durum wheat Langdon and *Ae*. *tauschii* accession PI 268210 (Joppa and Williams 1982). ND495 was developed at North Dakota State University (Fargo, ND, USA) and has a pedigree of Justin*2/3/ND 259/Conley//ND 112 (Anderson *et al.* 1999). The RILs along with parental lines ND495, Largo, PI 268210, and Langdon were phenotyped for seedling resistance in two biological replications (5 plants/replication) with *Pgt* races TTKSK (04KEN156/04), TRTTF (06YEM34-1), and TTTTF (01MN84A-1-2). The virulence/avirulence details of the three races are listed in Table 1.

**Table 1:** Avirulence and virulence profile of three *Puccinia graminis* f. sp. *tritici* (*Pgt*) races TTKSK, TRTTF, and TTTTF for the North American differentials

*Pgt* race (isolate)	Avirulent	Virulent
TTKSK (04KEN156/04)	*Sr24 36 Tmp*	*Sr5 6 7b 8a 9a 9b 9d 9e 9g 10 11 17 21 30 31 38 McN*
TRTTF (06YEM34-1)	*Sr8a 24 31*	*Sr5 6 7b 9a 9b 9d 9e* ^a^ *9g 10 11 17 21 30 36 38 McN Tmp*
TTTTF (01MN84A-1-2)	*Sr24 31*	*Sr5 6 7b 8a 9a 9b 9d 9e 9g 10 11 17 21 30 36 38 McN Tmp*

a Virulence of TRTTF to *9e* is variable due to the minor effect.

The stem rust screening experiment was performed under controlled greenhouse conditions at the USDA-ARS Cereal Disease Laboratory, St. Paul, MN using the procedure described by Hundie *et al.* (2019). Briefly, the primary leaves of the seedling plants at 7 to 9 days after planting were inoculated with the *Pgt* urediniospores. After inoculation, the plants were moved into a greenhouse maintained at 18 ± 2°C with a 16 hours photoperiod. The plants were scored for infection type (IT) at 14 days post inoculation based on the Stakman *et al.* (1962) 0–4 scale followed by the additional symbols (^+^ and ^-^) for the pustule size (Roelfs and Martens 1988). To identify the regions harboring quantitative trait loci (QTL) associated with resistance to the three *Pgt* races, the IT scores of each RIL for individual races were converted to the linearized IT (LIT) scores in a 0–9 scale as described by Zhang *et al.* (2014), where a score of 0 to 5 was considered as resistant and 6 to 9 considered as susceptible. To determine the repeatability of stem rust test for the RIL population, we conducted correlation analysis using LIT scores between two reps for each race. The *t*-tests (least significant difference) were also conducted to detect the RIL lines that significantly differ from the parents. The statistical analysis was conducted by using the PROC GLM procedure in SAS version 9.4 (SAS Institute Inc., Cary, NC, USA). The mean of the linearized IT (LIT) scores of two replications were used for the development of histograms and QTL analysis.

### Genotypic analysis

Out of 226 RILs used for the phenotypic analysis, 188 RILs were randomly selected for the genotypic analysis to avoid the bias in the marker data set. DNA extraction of 188 RILs along with parental lines ND495, Largo, PI 268210, and Langdon was done according to the procedure described in Faris *et al.* (2000). For genotyping, the wheat Infinium 90 K SNP iSelect array (Wang *et al.* 2014) was used and whole-genome linkage maps were developed by using the MapDisto 1.8.2.1 software package (Lorieux 2012) with a logarithm of odds (LOD) cut-off value of 3.0, and mapping distances were measured using the Kosambi mapping function (Kosambi 1943). The order of steps used for the linkage map development was followed as described in Sharma *et al.* (2019a). Briefly, linkage groups were first identified and then followed by fixing the marker order within each group by using the command “order sequence.” Next, “check inversions,” “ripple order,” and “drop locus” commands were used to generate robust linkage maps. For purposes of generating figures, linkage maps with few non-redundant loci were developed by using the software Mapchart 2.32 (Voorrips 2002).

### QTL analysis

To detect genomic regions associated with stem rust resistance, a QTL analysis was conducted using QGENE (4.3.10) software (Joehanes and Nelson 2008) and the single-trait multiple interval mapping (MIM) method (Kao *et al.* 1999). Based on the MIM statistical model (Kao *et al.* 1999), we assumed that there are *m* QTL (*Q_1_*, *Q_2_*, … *Q_m_*) for controlling resistance to stem rust in the RIL population*.* The resistance phenotype value *Y* for a RIL, *i*, can be related to the *m* putative QTL by the model (Kao *et al.* 1999).

$$Y_{i}=µ+\sum_{j=1}^{m}a_{j}x_{ij}+\sum_{j\neq k}^{m}\delta_{jk}(w_{jk}x_{ij}x_{ik})+\varepsilon_{i,}$$

where *µ* is the mean, *x_ij_* is coded as ½ (*Q_j_Q_j_*) or -½ (*q_j_q_j_*) for the genotype of *Q_j_*, *a_j_* is the main effect of *Q_j_*, and *w_jk_* is the epistatic effect between *Q_j_* and *Q_k_*, *δ_jk_* is the indicator for epistasis between *Q_j_* and *Q_k_*, and *ε_i_* is the error that is assumed to follow N(0, σ^2^). The LOD value 3.0 was set as the cut-off for the QTL detection. After identification of the gene-associated regions, simple sequence repeat (SSR) markers from marker sets BARC (Song *et al.* 2005), CFD (Guyomarc’h *et al.* 2002; Sourdille *et al.* 2003; Somers *et al.* 2004), WMC (Somers *et al.* 2004), and GWM (Röder *et al.* 1998) were further used to map the specific chromosomes. Four (*cfd15*, *cfd61*, *cfd72*, and *wmc429*) and three (*barc18*, *gwm388*, and *wmc154*) SSR markers were mapped on chromosomes 1 D and 2B, respectively. For the *Sr46* gene region, 10 previously known SSRs (*barc124*, *barc95*, *cfd36*, *cfd43*, *gwm102*, *gwm210*, *gwm261*, *gwm455*, *wmc112*, and *wmc25*) were mapped on chromosome arm 2DS. In addition, three SSRs (*Xrwgs46, Xrwgs47*, and *Xrwgs49*) developed based on reference genome sequences were also mapped (Table 2). The primers of these markers were designed using the Primer-BLAST suite (http://www.ncbi.nlm.nih.gov/tools/primer-blast/, last accessed June 15, 2021 ) based on sequences within the *Sr46* region of chromosome arm 2DS in *Ae tauschii* (AL 8/78) and hexaploid wheat (Chinese Spring) (IWGSC 2018). The SSR genotyping assays were performed using 6% non-denatured poly-acrylamide gels as described in Saini *et al.* (2018).

**Table 2:** The newly developed simple sequence repeat (SSR) markers mapped on the chromosome arm 2DS in ND495 × Largo recombinant inbred line population

Marker name	Primer name	primer sequence^a^	Tm (°C)	Position_length in reference genomes^b^	
				AL 8/78	Chinese Spring
*Xrwgs46*	Xrwgs46F Xrwgs46R	[Tail1]TGGAGCAAGCTAGTAGGGTT GATGCTCTTAGGTGACAACTC	58.05 56.08	7.178 M_152 bp	8.601 M_148 bp
*Xrwgs47*	Xrwgs47F Xrwgs47R	[Tail1]ATCACCGCTGCTAGTTCTTG CAAAGTCGAAGGGTAGAGCA	57.98 57.26	6.535 M_266 bp	7.640 M_415 bp
*Xrwgs49*	Xrwgs49F Xrwgs49R	[Tail1]GGACTGTTGTTGTTCGGTAC TGTACTTGGGTGTTTGGAGG	56.69 57.35	8.870 M_212 bp	10.065 M_173 bp

a Tail1 = GCAACAGGAACCAGCTATGAC-3′.

b Position coordinates and length on the *Aegilops tauschii* (AL8/78) and Chinese Spring reference genomes (IWGSC 2018).

### Data availability

The plant materials are available upon request. All data necessary for confirming the conclusions of the article are present within the article, figures, tables, and supplementary files. Supplemental material is provided at figshare: https://doi.org/10.25387/g3.14450454. Supplementary File S1 contains IT and LIT scores of all lines. Supplementary Files S2–S4 present the results of LSD tests for mean LIT scores of RILs and their parental lines tested with *Pgt* race TTKSK, TRTTF, and TTTTF, respectively. Supplementary File S5 contains whole-genome linkage maps and Supplementary File S6 contains genotypic data for 188 RILs genotyped with wheat Infinium 90 K SNP iSelect array and SSR markers.

## Results

Stem rust screening showed that Largo exhibited low infection types to *Pgt* races TTKSK (IT 2), TRTTF (ITs ;2^−^ and ;12^−^ in replicates 1 and 2, respectively), and TTTTF (IT 22^−^) (Table 3). ND495 was susceptible to TTKSK (IT 3^+^), moderately susceptible to TRTTF (ITs 31^+^ and 1^+^3^−^ in replicates 1 and 2, respectively), and resistant to TTTTF (IT ;3 and 0;13 in replicates 1 and 2, respectively). The Pearson correlation coefficients between the two replications for TTKSK, TRTTF, and TTTTF were 0.90, 0.83, and 0.95, respectively, which were highly significant (*P *<* *0.0001), indicating high repeatability of the two replicates in the tests with each race. Therefore, the mean linearized infection type (LIT) scores from both reps for each race were used in the subsequent analysis.

**Table 3:** **Infection types scored on ND495, Largo, and parents of Largo tested using races TTKSK, TRTTF, and TTTTF of stem rust pathogen (*Puccinia graminis* f. sp. *tritici*)** ^a^

Line	TTKSK		TRTTF		TTTTF	
	Rep 1	Rep 2	Rep 1	Rep 2	Rep 1	Rep 2
ND495	3^+^	3^+^	31^+^	1^+^3^−^	;3	0;13^−^
Largo	2	2	;2^−^	;12^−^	22^−^	22^−^
PI 268210	2^−^	2^−^	2^−^	2^−^	2^−^	2^−^;
Langdon	22^+^	22^+^	;2^−^	;2^−^	2^−^	2^−^

a Infection types (Its) were scored based on the Stakman *et al.* (1962) where 0, ;, 1, or 2, are considered resistant, and 3 or 4 are considered susceptible. For leaves exhibiting combinations of ITs, order indicates predominant types. Symbols “−” and “+” indicated small or large pustules, respectively, within a class.

**Figure 1:**
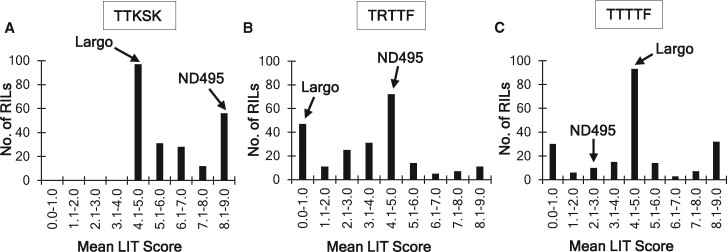
Histograms representing the mean distribution of the linearized infection type (LIT) score of two replications for each recombinant inbred line (RIL) of the ND495 × Largo population against the races TTKSK (A), TRTTF (B), and TTTTF (C) of *Puccinia graminis* f. sp. *tritici*.

The mean LIT scores of the RIL population for race TTKSK ranged from 4.5 to 9.0, with Largo and ND495 scoring 5.0 and 9.0, respectively (Figure 1A and Supplementary Files S1 and S2). There was no significant transgressive segregation detected even though one line (NL025) had slightly increased levels of resistance over Largo (Supplementary File S2). For TRTTF, the RILs had mean LIT scores that ranged from 0.0 to 9.0 with Largo and ND495 scoring 1.0 and 5.0, respectively (Figure 1B). Twelve lines showed increased levels of resistance (mean LITs 0.0 and 0.5) over Largo, but the increases were not significant (*P *≤* *0.05). However, 22 RILs (mean LITs 7.0–9.0) were significantly more susceptible than ND495 (Supplementary File S3), indicating the presence of transgressive segregation in the population. For reactions to TTTTF, the RIL population had mean LIT scores also ranging from 0.0 to 9.0 even though both parents were in the resistant range, with ND495 being more resistant than Largo (Figure 1C and Supplementary Files S1 and S4). In the population, 35 lines (mean LITs 0.0–1.0) had significantly lower mean LIT scores than ND495 (2.5), whereas 44 lines (6.5–9.0) had significantly higher mean LIT scores than Largo (5.0), indicating a strong transgressive segregation for resistance to TTTTF in the population.

**Figure 2:**
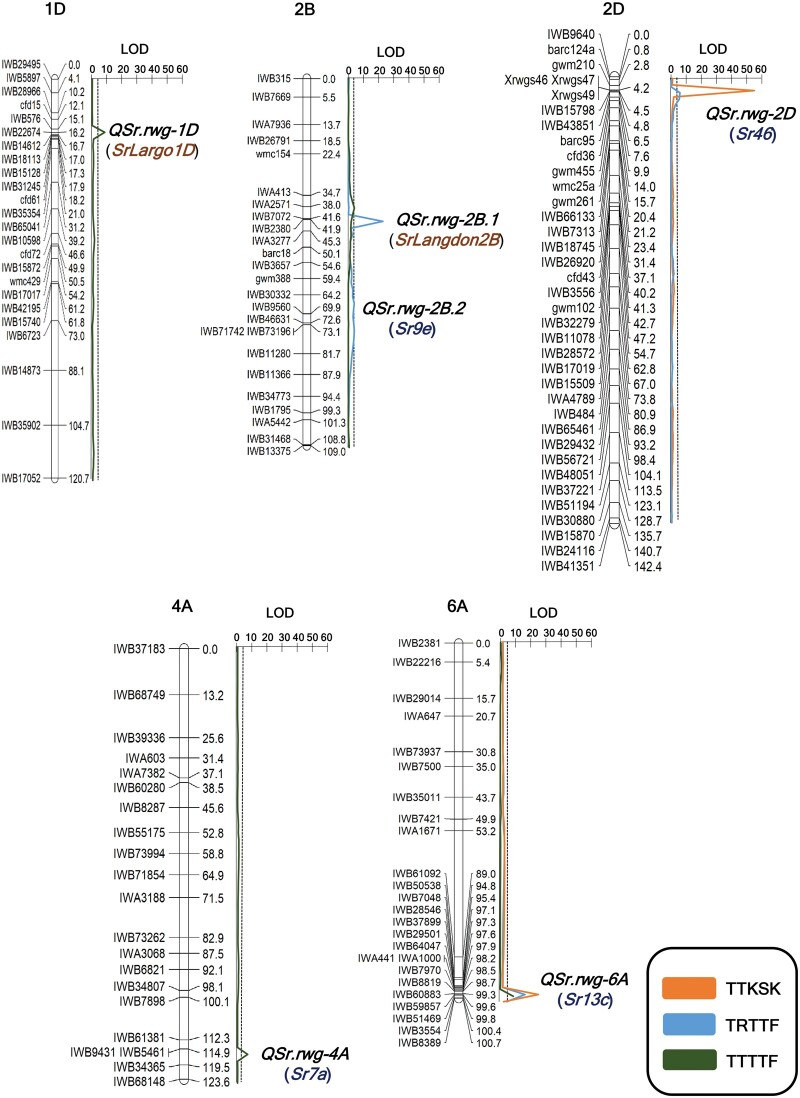
QTL regions identified across five chromosomes in the ND495 × Largo recombinant inbred line (RIL) population against the races of TTKSK, TRTTF, and TTTTF of *Puccinia graminis* f. sp. *tritici*. The critical LOD threshold is indicated by the *dotted line*. The confirmed stem rust resistance genes (*Sr*) associated with QTL regions are presented in brackets and *blue font*. The temporarily designated genes are presented in orange color.

Linkage maps were developed for the entire genome with 8203 (90 K SNP + SSR) markers representing 1739 loci across the 21 chromosomes and map density ranging from 0.9 cM/locus for chromosome 1B to 4.4 cM/locus for chromosome 4D (Table 4 and Supplementary Files S5 and S6). Two QTL regions associated with TTKSK resistance were identified on chromosome arms 2DS and 6AL designated as *QSr.rwg-2D* and *QSr.rwg-6A*, respectively (Table 5 and Figure 2). The *QSr.rwg-2D* QTL was positioned at 4 cM, flanked by *Xrwgs46* and *IWB43851* with a LOD value of 52.4 and explained 62.1% of the phenotypic variation (*R*^2^ × 100). This region of chromosome arm 2DS is known to carry *Sr46*. The second TTKSK-associated QTL, *QSr.rwg-6A*, was positioned at 98 cM and flanked by *IWA441* and *IWB51469*. It had a LOD value of 23.5 and explained 21.3% of phenotypic variation. The gene *Sr13* is known to lie within this genomic region. Both *QSr.rwg-2D* and *QSr.rwg-6A* had positive additive values of 1.2 and 0.7, respectively, indicating that TTKSK resistance was derived from Largo.

**Table 4:** The linkage maps developed in ND495 × Largo recombinant inbred line (RIL) population, number of markers, loci, and other genetic parameters

Chromosome	No. of markers			Loci	Length (cM)	cM/locus
	SSR	SNP	Total			
1A	—	662	662	77	80.3	1.0
1B	—	750	750	96	87.8	0.9
1D	4	258	262	69	120.7	1.7
2A	—	425	425	79	124.3	1.6
2B	3	663	666	77	109.0	1.4
2D	13	140	153	84	142.4	1.7
3A	—	442	442	97	124.2	1.3
3B	—	775	775	155	145.2	0.9
3D	—	164	164	58	120.2	2.1
4A	—	394	394	88	123.6	1.4
4B	—	426	426	68	83.3	1.2
4D	—	54	54	26	114.9	4.4
5A	—	515	515	155	155.9	1.0
5B	—	613	613	104	130.6	1.3
5D	—	150	150	76	123.9	1.6
6A	—	399	399	55	101.0	1.8
6B	—	478	478	61	83.7	1.4
6D	—	131	131	59	122.3	2.1
7A	—	270	270	79	131.5	1.7
7B	—	289	289	90	87.1	1.0
7D	—	185	185	86	146.9	1.7
A genome	—	3,107	3,107	630	840.8	1.3
B genome	3	3,994	3,997	651	726.7	1.1
D genome	17	1,082	1,099	458	891.3	1.9
Total	20	8,183	8,203	1,739	2,458.7	1.4

**Table 5:** Quantitative trait loci (QTL) identified in the ND495 × Largo recombinant inbred line population tested with races TTKSK, TRTTF, and TTTTF of stem rust pathogen

QTL	Putative gene	Flanking markers	Chr.^a^	Pos.^b^	TTKSK			TRTTF				TTTTF		
					LOD	Add.^c^	*R* ^2^ ×100		LOD	Add.	*R* ^2^ × 100	LOD	Add.	*R* ^2^ × 100
*QSr.rwg-1D*	*^d^	*IWB22674—IWB31245*	1D	16	—^e^	—	—		—	—	—	7.8	0.9	16.8
*QSr.rwg-2B.1*	*	*IWA413—IWA2571*	2B	38	—	—	—		—	—	—	3.0	0.5	4.6
*QSr.rwg-2B.1*	*	*IWB7072—IWB2380*	2B	42	—	—	—		22.9	1.3	33.3	—	—	—
*QSr.rwg-2B.2*	*Sr9e*	*IWB71742—IWB73196*	2B	74	—	—	—		4.0	0.5	16.2	—	—	—
*QSr.rwg-2D*	*Sr46*	*Xrwgs46—IWB43851*	2D	4	52.4	1.2	62.1		3.5	0.4	3.6	—	—	—
*QSr.rwg-4A*	*Sr7a*	*IWB9431—IWB5461*	4A	114	—	—	—	—	—	—	—	6.6	−0.8	16.1
*QSr.rwg-6A*	*Sr13c*	*IWA441—IWB51469/IWB25644*	6A	98-100	23.5	0.7	21.3		13.8	0.9	18.5	8.7	0.9	17.0

a Chr. = Chromosome.

b Pos. = Position in centimorgan (cM).

c Add. = Additive effect of the QTL, positive values indicate resistance derived from Largo and negative values indicates resistance derived from ND495.

d Symbol “*” indicates no known stem rust resistance gene.

e Symbol “—“ indicates no QTL identified.

For TRTTF, four QTL were identified on chromosome arms 2BS, 2BL, 2DS, and 6AL, designated as *QSr.rwg-2B.1*, *QSr.rwg-2B.2, QSr.rwg-2D*, and *QSr.rwg-6A*, respectively (Table 5 and Figure 2). *QSr.rwg-2B.1* (42.0 cM) was flanked by *IWB7072* and *IWB2380* with a LOD value of 22.9 and it explained 33.3% of the phenotypic variation. The second TRTTF-specific QTL, *QSr.rwg-2B.2* (74.0 cM), was identified on chromosome arm 2BL and was flanked by *IWB71742* and *IWB73196*. This QTL had a LOD value of 4.0 and explained 16.2% of phenotypic variation. The third TRTTF-associated QTL, *QSr.rwg-2D* (LOD = 3.5), was located on chromosome arm 2DS and was similar to the TTKSK QTL located in the *Sr46* region, however, its effect for TRTTF was less compared with TTKSK with an explained 3.6% of phenotypic variation. Likewise, the fourth QTL, *QSr.rwg-6A*, also coincided with the TTKSK and TRTTF QTL and explained 18.5% of the phenotypic variation for TRTTF resistance and had a LOD value of 13.8. The positive additive values for all the QTL regions suggest that resistance was derived from Largo (Table 5).

A total of four QTL were identified for resistance to *Pgt* race TTTTF on chromosome arms 1DS, 2BS, 4AL, and 6AL and were designated as *QSr.rwg-1D*, *QSr.rwg-2B.1*, *QSr.rwg-4A*, and *QSr.rwg-6A*, respectively (Table 5 and Figure 2). Among these four QTL, only the *QSr.rwg-4A* associated resistance was derived from ND495, whereas all others were derived from Largo. The *QSr.rwg-1D* (LOD = 7.8) was located at 16.0 cM and flanked by *IWB22674* and *IWB31245*, explaining 16.8% of phenotypic variation. *QSr.rwg-2B.1* was located at 38 cM and flanked by *IWA413* and *IWA2571.* This QTL explained 4.6% of phenotypic variation and was adjacent to the TRTTF QTL located at 42 cM on chromosome arm 2BS. *QSr.rwg-4A* (LOD = 6.6), which explained 16.1% of the phenotypic variation, was identified on chromosome arm 4AL at 114 cM and flanked by *IWB9431* and *IWB5461* located in the region known to be associated with *Sr7*. The *QSr.rwg-6A* region was common among the three *Pgt* races tested in this study, and for TTTTF it has maximum LOD at position 100 cM (distorted from TTKSK and TRTTF QTL peak). It explained 17.0% of the phenotypic variation and had a LOD value of 8.7.

## Discussion

Synthetic hexaploid wheat line Largo and its parents (Langdon and *Ae*. *tauschii* accession PI 268210) were previously reported to be resistant to multiple races of the stem rust pathogen (Salazar and Joppa 1981; Friesen *et al.* 2008; Zhang 2013). In the present study, we identified six major QTL (*QSr.rwg-1D*, *QSr.rwg-2B.1*, *QSr.rwg-2B.2*, *QSr.rwg-2D*, *QSr.rwg-4A*, and *QSr.rwg-6A*) on chromosomes 1DS, 2BS, 2BL, 2DS, 4AL, and 6AL, respectively, using the ND495 × Largo RIL population, suggesting that it segregated for at least six *Sr* genes. With the exception of *QSr.rwg-4A*, which was derived from the susceptible parent ND495, all the resistance QTL were derived from Largo.

The TTTTF-effective QTL *QSr.rwg-1D* was identified on the short arm of chromosome 1D (16 cM), located proximal to SSR marker *cfd15* (Figure 2 and Supplementary File S5). Thus far, three Ug99-effective *Sr* genes, *Sr33*, *Sr45*, and *SrTA1662*, have been identified on chromosome arm 1DS from *Ae. tauschii* (Sambasivam *et al.* 2008; Olson *et al.* 2013; Periyannan *et al.* 2013, 2014a; Arora *et al.* 2019). The *Sr33* gene is flanked by SSR markers *barc152* and *cfd15*. *SrTA1662* also mapped in the same region (Olson *et al.* 2013), while *Sr45* is positioned proximal to *Sr33*. Based on the location of SSR marker *cfd15*, *QSr.rwg-1D* mapped in the *Sr45* region, however, it was not effective against TTKSK. Therefore, phenotypic characterization of *QSr.rwg-1D* suggests that the gene underlying the *QSr.rwg-1D* is most likely different from *Sr33*, *Sr45*, and *SrTA1662* and further evaluation of this region is required.

The QTL *QSr.rwg-2B.1* was located near the centromeric region of chromosome arm 2BS and was effective against TRTTF and TTTTF. There are three *Sr* genes that have been reported to reside in this region and they include *Sr20*, *Sr36*, and *Sr40* (McIntosh *et al.* 1995). *Sr20* is not effective against TRTTF (Y. Jin, unpublished) and *Sr36* was reported to be ineffective against TRTTF (Olivera *et al.* 2012) and TTTTF (Jin and Singh 2006). Because *Sr40* is effective against TTKSK (Singh *et al.* 2015), the possibility that it is the gene underlying *QSr.rwg-2B.1* can be ruled out. In addition, both *Sr36* and *Sr40* are located on the alien chromosome segments 2 G#1S and 2 G#2S, respectively, which are derived from *T. timopheevii* (Zhuk.) Zhuk (2*n* = 4*x* = 28, AAGG) (Allard and Shands 1954; Nyquist 1957, 1962; Dyck 1992; Friebe *et al.* 1996), and *T. timopheevii* is not present in the pedigree and parentage of Langdon (Heyne 1959). Therefore, the *Sr* gene associated with *QSr.rwg-2B.1* is different from any known gene in this region, indicating a minor-effect *Sr* gene present in 2BS that originates from Langdon.

For the three *Pgt* races used in this study, the QTL *QSr.rwg-2B.2* located on chromosome arm 2BL was only effective against TRTTF (Figure 2). There have been several *Sr* genes reported on chromosome arm 2BL (*Sr9*, *Sr16*, *Sr28*, and *Sr883-2B*) (McIntosh 1995; Hiebert *et al.* 2010; Sharma *et al.* 2019b). For *Sr9*, seven alleles have been identified: *Sr9a*, *Sr9b*, *Sr9d*, *Sr9e*, *Sr9f*, *Sr9g*, and *Sr9h* (Green *et al.* 1960; Knott 1966; McIntosh and Luig 1973; Loegering 1975; Rouse *et al.* 2014). Among all these reported genes and their alleles, *Sr9e* is known to be present in many durum wheat varieties including Langdon (Luig 1983; Singh *et al.* 1992), and it has a minor effect against TRTTF (Saini *et al.* 2018). *Sr16* is not effective against TRTTF (Singh *et al.* 2015), and the *Sr28* gene is known to confer resistance against TTKSK, but *QSr.rwg-2B.2* did not condition resistance to this race. Based on the consensus map location of SNPs, the *Sr883-2B* gene reported by Sharma *et al.* (2019b) is located some distance from the *QSr.rwg-2B.2* (Wang *et al.* 2014). *Sr9h* is effective against *Pgt* race TTKSK (Singh *et al.* 2015), but *QSr.rwg-2B.2* resistance was not associated with TTKSK and TTTTF. Because Largo carries the TRTTF-effective gene *Sr9e* from Langdon and the other genes known to reside on 2BL can essentially be ruled out, it is most certain that the *Sr* gene underlying *QSr.rwg-2B.2* is *Sr9e*.

The TTKSK- and TRTTF-specific QTL *QSr.rwg-2D* was located near the distal end of the chromosome arm 2DS, which is a region known to harbor *Sr32* (Mago *et al.* 2013) and *Sr46* (Yu *et al.* 2015; Arora *et al.* 2019). Both genes are effective against TTKSK and TRTTF (Olivera *et al.* 2012). The *Sr32* gene was originally derived from *Ae. speltoides* Tausch (Friebe *et al.* 1996) and should not be the gene underlying the QTL *QSr.rwg-2D* because this gene had not been introduced into any of the parental lines (*i.e.*, Langdon, ND495 and Largo). *QSr.rwg-2D* was located proximal to *gwm210* and distal to *cfd36* (Figure 2 and Supplementary File S5), which corresponds to the *Sr46* location based on the map developed in the F_2_ population derived from the *Ae*. *tauschii* cross CIae 25 × AL8/78 (Yu *et al.* 2015), suggesting that *Sr46* is likely the gene underlying *QSr.rwg-2D*. *Sr46* is effective against TTKSK, TRTTF, and TTTTF (Yu *et al.* 2015), however, in the current analysis the *QSr.rwg-2D* was not associated with the TTTTF resistance. Based on this phenotypic difference, we speculate that the *Sr* gene underlying *QSr.rwg-2D* derived from Largo may be a different allele of *Sr46*. However, *Sr46* was mapped using the diploid *Ae*. *tauschii* F_2_ population, whereas the *QSr.rwg-2D* was identified in the hexaploid RIL population. Because genomic interaction in allopolyploid wheat often causes the reduction or suppression of resistance of some *Sr* genes (Hiebert *et al.* 2020), it is also possible that the different reactions of *Sr46* and *QSr.rwg-2D* to TTTTF were caused by different ploidy levels. Therefore, further study is needed to determine the identity of the gene for *QSr.rwg-2D*.

Among all the QTL identified in this study, only *QSr.rwg-4A* positioned on chromosome arm 4AL was derived from ND495 (Figure 2). This QTL conditioned resistance against *Pgt* race TTTTF and was located in the physical region known to be associated with the *Sr7* locus and a TTKSK-effective gene *SrND643* (Basnet *et al.* 2015; Saini *et al.* 2018). Because *QSr.rwg-4A* is not resistant to TTKSK, *SrND643* is apparently not the candidate gene for *QSr.rwg-4A*. To date, two alleles, *Sr7a* and *Sr7b*, have been reported at the *Sr7* locus (McIntosh *et al.* 1995). *Sr7b* is not effective against TTTTF, whereas *Sr7a* is effective against TTTTF and it is nearly fixed in the wheat breeding germplasm in the Northern Great Plains (Jin *et al.* 2007; Turner *et al.* 2016; Saini *et al.* 2018). As *QSr.rwg-4A* was located to the *Sr7* region and has resistance to TTTTF, most likely *Sr7a* is the underlying gene for this region.

The QTL *QSr.rwg-6A* derived from Langdon is located on chromosome arm 6AL, which carries three known TTKSK-effective *Sr* genes, *Sr13*, *Sr26*, and *Sr52*. Among these known genes, *Sr26* and *Sr52* were originally transferred into wheat from wild species *Thinopyrum ponticum* (Podp.) Barkw. & D.R. Dewey [*Agropyron elongatum* (Host) Beauv.] (Knott 1961; Dundas *et al.* 2007) and *Dasypyrum villosum* (L.) Candargy (Qi et *al*. 2011), respectively. Because *Sr26* and *Sr52* have not been transferred into durum wheat Langdon, they can be ruled out as the causal gene for *QSr.rwg-6A*. This QTL was known to be physically associated with *Sr13* and effective against all three *Pgt* races used in the current analysis (McIntosh 1995; Simons *et al.* 2011; Periyannan *et al.* 2014b; Zhang *et al.* 2017; Gill *et al.* 2021). Gill *et al.* (2021) identified *Sr13* as the causal gene for the stem rust resistance in an accession PI 387696 of *T. turgidum* subsp. *carthlicum* (Neyski) Á. Löve & D. Löve. By comparing the *QSr.rwg-6A* region to the *Sr13* region in the study by Gill *et al.* (2021), we found that six SNP markers (*IWB61092*, *IWB50538*, *IWB7048*, *IWB28546*, *IWB37898*, and *IWB34398)* in the two regions were in common in both 90 K SNP-based high-density maps. Two of the markers, *IWB37898* and *IWB34398*, that are tightly linked to *Sr13* in the study by Gill *et al.* (2021) are also located in the *QSr.rwg-6A* region. Zhang *et al.* (2017) identified *Sr13* as a coiled-coil nucleotide-binding leucine-rich repeat (NLR) gene. They identified three resistant (R1–R3) and 10 susceptible (S1–S10) haplotypes of this gene based on the reactions to TTKSK and designated R1/R3 and R2 as *Sr13a* and *Sr13b*, respectively, based on their resistant and susceptible reactions to JRCQC. Gill *et al.* (2021) re-designated the R1 and R3 haplotypes as *Sr13a* and *Sr13c*, respectively, based on their susceptible and resistant reactions to TCMJC. Among different diploid, tetraploid, and hexaploid wheat accessions that have been characterized for these haplotypes, Langdon was categorized as having the R3 haplotype of *Sr13* (Zhang *et al.* 2017; Gill *et al.* 2021). Because Langdon is present in the Largo background, *Sr13c* is the gene underlying *QSr.rwg-6A*.

In summary, we mapped three known *Sr* genes *Sr9e* (*QSr.rwg-2B.2*), *Sr13c* (*QSr.rwg-6A*), and *Sr7a* (*QSr.rwg-4A*) in the ND495 × Largo RIL population. In addition, there were three other genomic regions associated with stem rust resistance. Of these three *Sr* regions, *QSr.rwg-1D* (likely a new gene, temporarily designated as *SrLargo1D*) and *QSr.rwg-2D* (possibly a new allele of *Sr46*) were derived from the *Ae*. *tauschii* parent of Largo. The *QSr.rwg-2B.1* derived from Langdon is located in a region with no known *Sr* genes. Therefore, *QSr.rwg-2B.1* is probably associated with a new *Sr* gene (temporarily designated as *SrLangdon2B*) against *Pgt* races TRTTF and TTTTF. As no evaluation was previously performed to identify the *Sr* gene(s) in ND495, identification of *Sr7a* (*QSr.rwg-4A*) in this study suggests that ND495 carries *Sr* gene(s) with minor effects. The identification of these *Sr* genes in Largo will guide the future efforts to stack multiple resistant genes. Among the Ug99-effective *Sr* genes, both *Sr13c* and *Sr46* had resistance to a broad spectrum of *Pgt* races. However, they are among a few genes from the primary gene pool that have not been utilized or deployed in modern bread wheat germplasm. Several NIL lines such as NL143, NL159, and NL193 with resistance to the three *Pgt* races were found to carry all the six genes, they may serve as the donors for simultaneously introducing *Sr13c*, *Sr46*, and four other genes into adapted bread wheat germplasm and varieties. Because Largo has resistance to other fungal diseases, the 90 K SNP marker data set and the high-density linkage map developed in this study will be useful for identifying and mapping the genes controlling other agronomically important traits derived from durum wheat (Langdon), bread wheat (*e.g.*, ND495), and *Ae*. *tauschii* (PI 268210).
